# Distinguishing Between Multisystem Inflammatory Syndrome, Associated With COVID-19 in Children and the Kawasaki Disease: Development of Preliminary Criteria Based on the Data of the Retrospective Multicenter Cohort Study

**DOI:** 10.3389/fped.2021.787353

**Published:** 2021-11-10

**Authors:** Mikhail M. Kostik, Liudmila V. Bregel, Ilia S. Avrusin, Elena A. Dondurei, Alla E. Matyunova, Olesya S. Efremova, Eugenia A. Isupova, Tatiana L. Kornishina, Vera V. Masalova, Ludmila S. Snegireva, Vladimir V. Shprakh, Yuri A. Kozlov, Olga V. Kalashnikova, Vyacheslav G. Chasnyk

**Affiliations:** ^1^Hospital Pediatry, Saint-Petersburg State Pediatric Medical University, Saint Petersburg, Russia; ^2^Irkutsk State Medical Academy of Postgraduate Education, Branch of Russian Medical Academy of Continuing Professional Education, Irkutsk, Russia; ^3^The First Russian Kawasaki Disease Center n.a. T. Kawasaki, Irkutsk Regional Children's Hospital, Irkutsk, Russia; ^4^Division of Pediatric Respiratory Infections, Scientific Research Institute of Influenza n.a. A.A. Smorodintsev, Saint Petersburg, Russia; ^5^Infection Diseases Department, Children's City Clinical Hospital #5 n.a. N.F. Filatov, Saint Petersburg, Russia

**Keywords:** multisystem inflammatory syndrome, Kawasaki disease, children, hypercytokine syndrome, cytokine storm syndrome, COVID-19, SARS-CoV-2

## Abstract

**Objectives:** Diagnostic between multisystem inflammatory syndrome associated with COVID-19 in children (MIS-C) and Kawasaki disease (KD) can make difficulties due to many similarities. Our study aimed to create a Kawasaki/MIS-C differentiation score (KMDscore) allowing discrimination of MIS-C and KD.

**Study design:** The retrospective multicenter cohort study included clinical, laboratory, and instrumental information about MIS-C (*n* = 72) and KD (*n* = 147). The variables allowed to discriminate both conditions used to construct and validate the diagnostic score called the KMDscore.

**Results:** Patients with MIS-C were older, had earlier admission to the hospital, had a shorter time before fever resolution, two times frequently had signs of GI and CNS involvement observed, and had more impressive thrombocytopenia, higher level of CRP, ferritin, ALT, AST, LDH, creatinine, triglycerides, troponin, and D-dimer compared to KD patients. Respiratory signs in MIS-C were presented with pleuritis, acute respiratory distress syndrome, oxygen dependency, lung infiltration, and ground-glass opacities in CT. The heart involvement with fast progression of myocarditis provided the severity of MIS-C and ICU admission due to 12 times higher arterial hypotension or shock and required cardiotonic. No differences in the frequency of CA lesions were seen in the majority of cases. Five criteria, CRP >11 mg/dl (18 points), D-dimer >607 ng/ml (27 points), age >5 years (30 points), thrombocytopenia (25 points), and GI involvement (28 points), were included in the KMDscore. The summa >55 points allowed to discriminate MIS-C from KD with a sensitivity of 87.5% and specificity of 89.1%.

**Conclusion:** The KMDscore can be used to differentiate the diagnostic of MIS-C from KD.

## Introduction

In December 2019, the first case of a new coronavirus infection with the severe acute respiratory syndrome (SARS-CoV-2) was registered in China for the first time. Since then, the COronaVIrus Disease 2019 (COVID-19) pandemic has rapidly developed into a global health emergency around the world.

This disease is observed relatively less often in children than in adults (1–5% of diagnosed diseases among the population at the beginning of the pandemic) (1). At the moment, it is up to 16% of all COVID-19 cases (2). The clinical course of COVID-19 in children, as in adults, mainly manifests with fever and respiratory symptoms; however, more frequently it proceeds in an asymptomatic or mild form, without development of severe pneumonia (3, 4). At the same time, since April 2020, there have been many reports that a new coronavirus infection may be associated with a childhood hyperinflammatory condition that fully or partially meets the criteria of Kawasaki disease (KD) (5).

KD is an acute systemic vasculitis of unknown etiology, affecting predominantly children under 5 years, characterized by fever, bilateral conjunctival hyperemia, oropharyngeal mucosa changes, erythematous rash, erythema and indurative palms and feet edema, and cervical lymphadenopathy. Approximately 20–25% of untreated patients develop changes in coronary arteries of varying severity from asymptomatic dilatation to giant aneurysms, thrombosis, myocardial infarction, and sudden death (6).

For the first time, the suspicion of the possible connection between COVID-19 and KD was put forward by Jones et al., who reported a case of classic KD in a 6-month-old girl with a positive PCR result for SARS-CoV-2 (7). Also among the first who described this problem were groups of researchers from Italy (8) and France (9). However, this disorder occurs in older children than KD. It is also often manifested with gastrointestinal symptoms (diarrhea, abdominal pain, vomiting) and heart damage (myocarditis, pericarditis), often leading to myocardial damage and shock, while these clinical manifestations are less common in KD (10). The severity and pronounced similarity of the symptoms of this new syndrome and KD caused terminological dilemmas—in addition to the name Kawasaki-like syndrome, others appeared—hyperinflammatory shock, Kawa-COVID, a multisystem inflammatory syndrome in children (MIS-C), or pediatric inflammatory, multisystem syndrome temporarily associated with SARS-CoV-2 (PIMS-TS), and since July–August 2020, two of the latter names have mainly been fixed in the literature. MIS-C is quite rare, and the incidence at the beginning of the pandemic in children was about 2 per 100,000 people or <1% of children with confirmed SARS-CoV-2 infection (11). However, in a more recent study, the frequency of MIS-C cases is 1:4,000 children who have suffered COVID-19 infection (12). Despite the similarities in the clinical picture, the diseases might have different outcomes and treatment approaches. Thus, KD required IVIG 2 g/kg as the first treatment line, followed by repeated IVIG, corticosteroids, and TNF-a inhibitors. MIS-C required systemic corticosteroids, IVIG, IL-1, and IL-6 inhibitors. Interestingly, anti-IL1 and IL-6 treatment did not show its efficacy in KD as anti-TNFa (13).

Our study is aimed to compare clinical and laboratory features of MIS-C and KD and to create the discrimination criteria between two diseases.

## Methods

### Patients

In the retrospective multicenter cohort study, we took the information from all medical records of patients who satisfied the criteria of MIS-C and KD at two university-affiliated tertiary hospitals in Russia and the biggest hospital for COVID-19-infected children (St. Petersburg, Irkutsk). We included all available cases of MIS-C (*n* = 72) from May 2020 to April 2021 and KD (*n* = 147) from September 2010 to February 2021. The diagnosis of MIS-C and KD was made according to the existing criteria (5, 14). We extracted the following medical information: demographics (age, sex), clinical features (highest recorded temperature, duration of fever, signs of involvement GI, CNS, respiratory and cardiovascular systems, presence of sore throat, rash, conjunctivitis, red dry, cracked lips, bright mucosa, cervical lymphadenopathy, distal extremity changes, peeling of the fingers, face swelling, hepatomegaly, splenomegaly and presence of arthritis), laboratory findings (complete blood count, ESR, ALT, AST, total protein, albumin, ferritin, LDH, CRP, triglycerides, creatinine, troponin I, fibrinogen, and D-dimer), treatment options, and transferring to ICU. Heart involvement meant the presence of any of the following: myocarditis (tachycardia, accompanied with at least one of the following signs: ECG and EchoCG changes, positive troponin I and/or BNP test), arterial hypotension/shock, pericarditis, or coronary artery (CA) lesions defined on EchoCG (5, 15). The laboratory parameters in the medical records were obtained on the peak of the disease (highest or lowest meanings). In MIS-C, we took epidemiological data about the presence of COVID-19 disease, type of identification (PCR throat or nasal swab, IgM, IgG), family or close contact, and time since the COVID-19 and MIS-C. For diagnostics of cytokine storm, we calculated HScore (16). For treatment of MIS-C and KD, we used the national and international treatment guidelines (5, 17–19).

### Ethics

Approval of the local ethical committee was not required since the study used data from clinical charts. All patients were appropriately anonymized. All patients' representatives and patients 15 years and older gave the consent in their case reports form allowing to use the medical information anonymously.

### Statistics

Sample size was not calculated initially. Statistical analysis was performed with the software STATISTICA, version 10.0 (StatSoft Inc., Tulsa, OK, USA). All continuous variables were checked by the Kolmogorov–Smirnov test, with no normal distribution identified. Continuous variables are presented as median and interquartile ranges (IQRs). Categorical variables are presented as proportions. Missing data were not imputed or included in the analyses. Pearson's χ^2^ test or the Fisher's exact test in the expected frequencies <5 was used to compare the categorical variables. A comparison of two quantitative variables was carried out using the Mann–Whitney test. The ability of each variable to discriminate MIS-C from KD was evaluated with sensitivity and specificity analysis, AUC-ROC (area under the receiver operating characteristic curve) with 95% confidence interval (CI), and calculation of the odds ratio (OR) for the detection of the best cutoffs of continuous variables. The higher values of OR of variables interfere with the better discriminatory ability. We used the “best” threshold for our data's ROC curve analysis because it provides the most appropriate mean between sensitivity and specificity. *p* < 0.05 was considered statistically significant. By univariate analysis, each of the variables of interest was associated with the positive diagnosis of MIS-C, with a *p* of < 0.05. They were therefore included in a multivariate logistic model to assess their independent contribution to the outcome. Binary variables included in the model (e.g., thrombocytopenia) were coded as present or absent. The threshold value was based on a ROC curve analysis, retaining the value at which sensitivity plus specificity was maximized. No interaction terms were included in the model. The pseudo R^2^ statistic was used for assessing the goodness of fit of the model. The coefficients resulting from this multiple logistic regression analysis were used to assign score points for the construction of the KMDscore. For each variable that was significantly associated with the outcome in the logistic regression, the rule was to multiply the beta value for each range by 100 and round off to the nearest integer.

## Results

### Epidemiology of COVID-19 in the Patients With MIS-C

COVID-19 infection was confirmed by throat/nasal swab SARS-CoV-2 PCR in 12/68 (17.7) at the moment of hospital admission due to MIS-C; both IgM and IgG antibodies against SARS-CoV-2 virus were positive in 25/62 (40.3%), while IgG only in 59/62 (95.2%). Close family contacts were identified in 33/72 (45.8). Twenty-two patients (30.6%) had symptomatic COVID-19 infection after family contacts, which were confirmed only clinically and epidemiologically. Patients had mild to moderate fever, anosmia, sneezing, and coughing. No cases of pneumonia or hospital admission were identified. Between COVID-19 infection or close family contact and MIS-C onset, the median time was 30.0 (21.0, 40.0) days.

### Clinical Differences Between MIS-C and KD

Patients with MIS-C had higher age and similarity in gender distribution with slight male predominance. Patients with MIS-C had earlier admission to the hospital and a shorter time before fever resolution.

Regarding clinical signs, patients with MIS-C had signs of GI (abdominal pain, vomiting, diarrhea, peritoneal signs) and CNS (irritability, headaches, seizures, aseptic meningitis) involvement two times more frequently. Approximately 65% of MIS-C patients admitted to ICU had CNS involvement. Kawasaki-associated signs were also frequent in MIS-C: sore throat, rash, conjunctivitis (hemorrhagic in MIS-C and non-purulent in KD), mucous involvement (bright lips and dry, cracked lips), distal edema, and face swelling. Respiratory disorders in MIS-C were presented with pleuritis, acute respiratory distress syndrome, oxygen dependency, lung infiltration, and ground-glass opacities in CT. The heart involvement was linked to the severity of MIS-C and ICU admission in the majority of cases. We have not seen differences in the frequency of CA lesions, but in MIS-C, the CA lesions were presented with mild or moderate CA dilatation and were reversible, compared to KD, where aneurysms (including giant ones) were. The frequency of pericarditis was of borderline significance. Myocarditis in MIS-C was characterized by fast progression (heart dilatation, decreasing LV ejection fraction, transient ECG changes—AV-blocks and repolarization disturbances) and was associated with arterial hypotension or shock, which required inotropic support with cardiotonic. Patients with heart involvement had increased troponin I, proBNP, CK, CK-MB, and LDH. Hypotension/shock with multiorgan failure accompanied with myocarditis and ARDS were the main reasons for ICU admission in both diseases, 12 times higher in MIS-C and only in a few patients with KD. Arterial hypotension/shock did not correlate with the degree of LV ejection fraction lowering. Data are shown in Table 1.

**Table 1:** Comparison of clinical and laboratory data between MIS-C and KD.

**Parameter**	**MIS-C (n = 72)**	**KD (n = 147)**	***p***
**Demographics**			
Age, years	8.9 (5.3, 11.8)	2.8 (1.0, 4.8)	0.0000001
Gender, male, n (%)	45 (62.5)	79 (53.7)	0.180
Days before hospital admission	5.0 (3.0, 9.0)	9.0 (5.0, 19.0)	0.000002
**Clinical signs**			
Duration of fever, days	11 (8, 13)	14 (9, 23)	0.001
Gastrointestinal symptoms, n (%)	58/71 (81.7)	58 (39.5)	0.0000001
Neurological symptoms, n (%)	31/69 (44.9)	34 (23.1)	0.001
Sore throat, n (%)	52/69 (75.4)	79 (53.7)	0.002
Rash, n (%)	59 (81.9)	108 (73.5)	0.166
Conjunctivitis, n (%)	64/69 (92.8)	108 (73.5)	0.001
Dry cracked lips, n (%)	42/66 (63.6)	42/95 (44.2)	0.015
Bright mucous, n (%)	52/69 (75.4)	93 (63.3)	0.078
Respiratory signs, n (%)	40 (55.6)	58 (39.5)	0.024
Cervical lymphadenopathy, n (%)	50/66 (75.8)	104 (70.8)	0.450
Hand/foot erythema/edema, n (%)	51/65 (78.5)	91 (61.9)	0.018
Peeling of fingers, n (%)	35/62 (56.5)	81 (55.1)	0.858
Face swelling, %	40/65 (61.5)	41/146 (28.1)	0.000004
Hepatomegaly, n (%)	50/70 (71.4)	100/133 (75.0)	0.327
Splenomegaly, n (%)	31/69 (44.9)	75/138 (54.3)	0.094
Arthritis/arthralgia, n (%)	17/69 (24.6)	39 (26.5)	0.767
CNS involvement, n (%)	31 (43.0)	34 (23.1)	0.001
Heart involvement, n (%)	51 (70.8)	73 (49.7)	0.003
Myocarditis, n (%)	34 (47.2)	34 (23.1)	0.0003
Pericarditis, n (%)	31 (43.1)	44 (29.9)	0.055
Coronary artery dilatation/aneurysm, n (%)	13 (18.1)	36 (24.5)	0.283
Hypotension/shock, n (%)	34 (47.2)	6 (4.1)	0.0000001
ICU admission, n (%)	37 (51.4)	12 (8.2)	0.0000001
**Laboratorial**			
Hemoglobin, g/l	103.5 (91.0, 113.0)	104.0 (94.0, 114.0)	0.558
White blood cells, 10^9^/l	15.8 (11.1, 20.9)	14.0 (8.7, 21.2)	0.360
Platelets, 10^9^/l	185 (95, 445)	520 (383, 666)	0.0000001
Thrombocytosis, n (%)	20/70 (28.6)	103/145 (71.0)	0.0000001
Thrombocytopenia, n (%)	36/70 (51.4)	7/144 (4.9)	0.0000001
ESR, mm/h	43 (28, 53)	48 (28, 62)	0.142
C-Reactive protein, mg/dl	16.3 (10.2, 24.2)	3.53 (1.0, 10.4)	0.0000001
Ferritin, μg/l	366 (209, 643)	120 (71, 239)	0.000004
Increased ferritin, n (%)	43/54 (79.6)	21/44 (47.7)	0.001
ALT, IU/l	46.0 (25.0, 78.0)	26.0 (14.9, 68.5)	0.003
Increased ALT, n (%)	38/70 (54.3)	47/140 (33.6)	0.004
AST, IU/l	53.0 (33.9, 87.0)	38.0 (30.0, 63.0)	0.0183
Increased AST, n (%)	46/55 (83.6)	52/112 (46.4)	0.000004
Serum protein, g/l	56.0 (49.0, 62.0)	68.9 (63.0, 75.8)	0.0000001
Albumin, g/l	30.6 (25.8, 34.0)	38.0 (33.0, 43.0)	0.0000001
Triglycerides, mmol/l	2.3 (1.8, 3.3)	1.0 (0.0, 2.1)	0.0001
Creatinine, mmol/l	57.2 (43.6, 71.4)	39.0 (34.3, 44.9)	0.0000001
LDH, IU/l	339 (248, 637)	291 (237, 357)	0.021
Increased LDH, n (%)	43/63 (68.3)	17/83 (20.5)	0.0000001
Troponin, pg/ml	10.0 (3.5, 4.2)	7.0 (2.0, 10.0)	0.016
Fibrinogen, g/l	4.6 (2.3, 6.5)	3.2 (2.5, 4.5)	0.097
D-Dimer, ng/ml	1,855 (938, 3,266)	584 (243,1,893)	0.001
HScore	112 (90, 142)	75 (68, 91)	0.0000001
**Treatment and outcomes**			
IVIG treatment, n (%)	37/67 (55.2)	127 (86.4)	0.000001
IVIG repeated course, n (%)	2/50 (4.0)	11/85 (12.9)	0.089
Acetyl salicylic acid, n (%)	42/67 (62.7)	128 (87.1)	0.00004
Corticosteroid treatment, n (%)	61/69 (88.4)	30 (20.4)	0.0000001
Biologics, n (%)	2/43 (4.7)	2/88 (2.3)	0.456
Stay in hospital, days	25 (18, 35)	18 (13, 24)	0.00001

*ALT, alanine aminotransferase; AST, aspartate aminotransferase; ESR, erythrocyte sedimentation rate; ICU, intensive care unit; IVIG, intravenous immunoglobulin; KD, Kawasaki disease; LDH, lactate dehydrogenase; MIS-C, multisystem inflammatory syndrome in children*.

### Laboratory Differences

Patients with MIS-C had more impressive thrombocytopenia and higher levels of CRP, ferritin, ALT, AST, LDH, creatinine, triglycerides, troponin, and D-dimer. Data are shown in Table 1.

### Creation of the Differentiation Model

**Figure 1:**
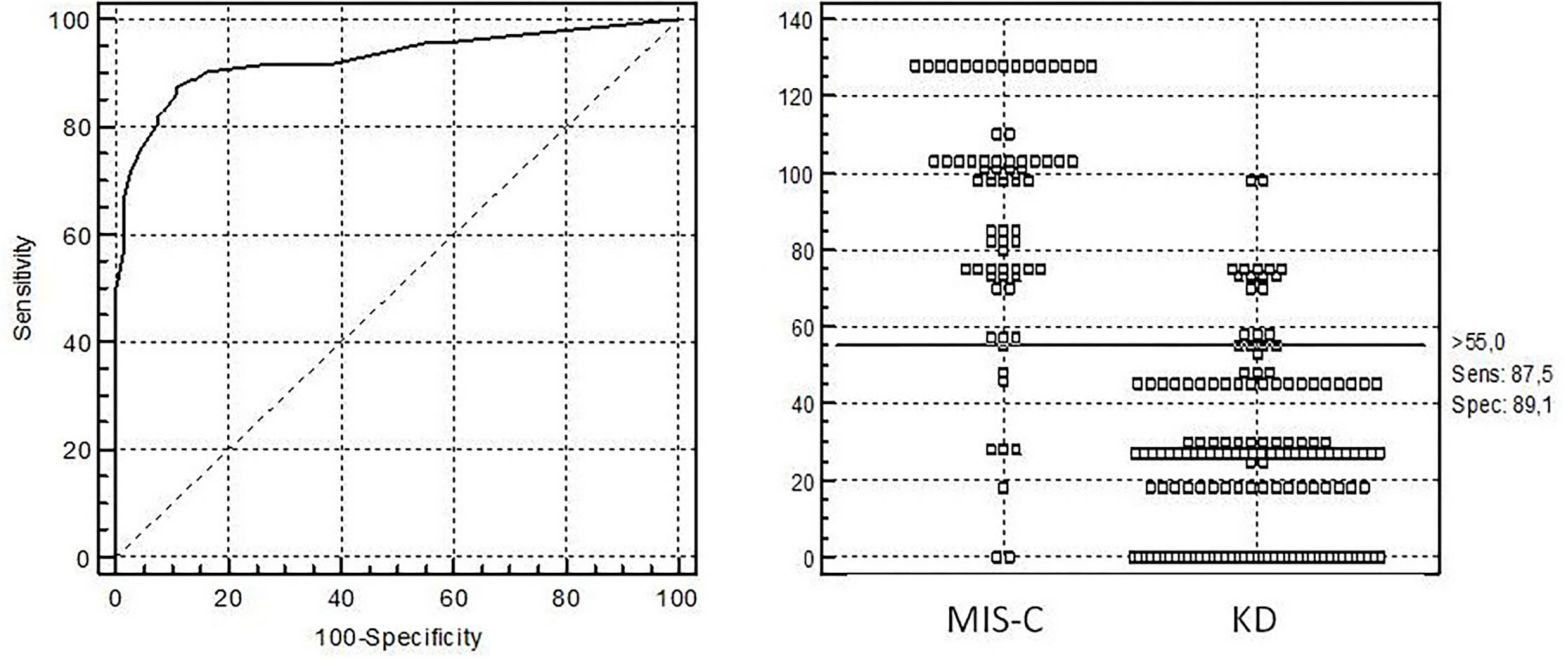
Receiver operating characteristic (ROC) curve analysis for diagnosis of multisystem inflammatory syndrome in children with diagnostic score (KMDscore) computed with the developmental data set. The optimal cutoff was selected as the threshold giving the highest value for the sum of sensitivity and specificity. Area under the curve (AUC) = 0.927 (0.884–0.958), DS for MIS-C >55 points with sensitivity of 87.5% and specificity of 89.1%.

In the next step, we selected continuous and categorical variables with statistical significance, and analysis of sensitivity and specificity with OR calculation was done. Data are shown in Table 2. Then, we extracted parameters with highest sensitivity, specificity, OR, and clinical meaningfulness. We excluded duplicated parameters, and multivariate analysis was allowed to extract five criteria: CRP >11 mg/dl, D-dimer >607 ng/ml, age >5 years, thrombocytopenia, and GI involvement. In the multivariate analysis, only five variables from the initial 29 included in the model remained significantly associated with the probability of being classified as having MIS-C. The optimal cutoff was selected as the threshold giving the highest value for the sum of sensitivity and specificity. The area under the curve (AUC) = 0.927 (0.884–0.958), DS for MIS-C >55 points, allowed to discriminate MIS-C from KD with sensitivity of 87.5% and specificity of 89.1% (Table 3; Figure 1). The pseudo R^2^ statistic for the model was 0.73 (*p* < 0.0001). The maximum possible score assigned to each variable varied from 18 for CRP > 11 mg/dl to 30 for age >5 years (Table 3). Missing data were scored as 0.

**Table 2:** Sensitivity, specificity, and odds ratios of clinical and laboratorial predictors, allowing to discriminate MIS-C and KD.

**Clinical predictors**	***Se***	***Sp***	**OR (95% CI)**	***p***
Age >5 years	76.1	83.5	16.0 (7.9, 32.4)	0.000001
Duration of fever <14 days	74.4	58.9	4.2 (1.9, 9.0)	0.0002
Respiratory signs	55.6	60.5	1.9 (1.1, 3.4)	0.024
Gastrointestinal signs	81.7	60.5	6.9 (3.5, 13.6)	0.0000001
CNS involvement	44.9	76.9	2.7 (1.5, 5.0)	0.001
Sore throat	75.4	46.3	2.6 (1.4, 5.0)	0.0024
Conjunctivitis	92.8	26.5	4.6 (1.7, 12.3)	0.001
Dry cracked lips	63.6	55.8	0.45 (0.24, 0.86)	0.015
Hands/feet erythema/edema	78.5	38.1	2.2 (1.1, 4.4)	0.018
Face swelling	61.5	71.9	4.1 (2.2, 7.6)	0.000004
Hypotension/shock, n (%)	47.2	95.9	21.0 (8.2, 53.8)	0.0000001
Any heart involvement, n (%)	55.2	13.6	2.5 (1.4, 4.5)	0.003
Myocarditis, n (%)	47.2	76.9	3.0 (1.6, 5.4)	0.0003
Pericarditis, n (%)	43.1	70.1	1.8 (0.99, 3.2)	0.055
ICU admission, n (%)	51.4	91.8	11.9 (5.6, 25.2)	0.0000001
**Laboratorial predictors**				
Platelets ≤ 264 × 10^3^ μl	62.9	88.7	13.2 (6.5, 26.9)	0.0000001
Thrombocytosis	28.6	29.0	0.16 (0.09, 0.31)	0.000000
Thrombocytopenia	51.4	95.1	20.7 (8.5, 50.6)	0.0000001
C-reactive protein > 11 mg/dl	74.6	79.1	11.1 (5.6, 22.3)	0.0000001
Ferritin > 260 ng/ml	70.4	79.2	9.0 (3.6, 22.4)	0.000001
Hyperferritinemia	79.6	52.3	4.3 (1.8, 10.4)	0.001
ALT > 22 U/l	82.9	46.8	4.3 (2.1, 8.6)	0.00003
Increased ALT	54.3	66.4	2.4 (1.3, 4.2)	0.004
AST > 50 U/l	55.1	66.9	2.5 (1.4, 4.5)	0.003
Increased AST	83.6	53.6	5.9 (2.6, 13.2)	0.000004
Serum protein ≤ 65 g/l	84.3	68.3	11.6 (5.5, 24.4)	0.0000001
Albumin ≤ 35 g/l	83.9	67.5	10.8 (4.7, 24.6)	0.0000001
Triglycerides > 1.38 mmol/l	32.0	12.5	14.9 (4.2, 52.4)	0.000004
Creatinine > 49.5 mmol/l	68.2	86.6	13.8 (6.1, 31.4)	0.0000001
Troponin > 25 pg/ml	5.1	58.8	13.0 (2.7, 62.8)	0.0002
D-Dimer > 607 ng/ml, n (%)	48.0	3.8	27.1 (5.9, 123.6)	0.0000001
Increased LDH, n (%)	68.3	79.5	3.3 (1.5, 7.4)	0.000000
HScore > 105, n (%)	56.5	76.2	4.2 (2.3, 7.7)	0.000002

*ALT, alanine aminotransferase; AST, aspartate aminotransferase; ICU, intensive care unit; IVIG, intravenous immunoglobulin; KD, Kawasaki disease; LDH, lactate dehydrogenase; MIS-C, multisystem inflammatory syndrome in children; OR, odds ratio; Se, sensitivity; Sp, specificity*.

**Table 3:** Variables included in the development of the diagnostic set and KMDscore calculation.

	**β**	**SE**	***p***		No. of points (criteria for scoring)^**^
CRP >11 mg/dl	0.18	0.73	0.016	CRP	0 (<11.0 mg/dl) or 18 (≥11.0 mg/dl)
D-Dimer >607 ng/ml	0.27	0.64	0.00006	D-dimer	0 (≤ 607 ng/ml) or 27 (>607 ng/ml)
Age >5 years	0.30	0.62	0.000004	Age	0 (≤ 5 years) or 30 (>5 years)
Thrombocytopenia^*^	0.2	0.62	0.0001	Platelets^*^	0 (no) or 25 (yes)
GI involvement	0.28	0.61	0.00001	GI involvement	0 (no) or 28 (yes)

* *Thrombocytopenia defined as platelets < 150 × 10^3^ μl.*

** *DS cutoff > 55 points*.

## Discussion

We are presenting the experience in diagnostics of MIS-C associated with COVID-19 at several pediatric clinics in Russia (St. Petersburg, Irkutsk) comparing them with a historical cohort of patients with KD. In general, the data obtained about both diseases are comparable with previously published studies. Special attention is focused on this new pathology due to the rapid development and high frequency of life-threatening complications—shock and multiorgan failure, as well as heart damage and coagulopathy (9, 11, 20). There are two sets of criteria for MIS-C diagnostics: the WHO criteria and CDC-criteria. We used WHO criteria, which might be to some extent focused on cardiac involvement, as CDC-criteria are more relevant to hyperinflammation, but all of our patients satisfied both sets of criteria (14, 21). Fortunately, MIS-C is a rare condition, which involves one of each 4,000 children, infected with COVID-19 (12). The obtained data demonstrate similarities and differences between MIS-C and KD. Both of these conditions are considered as hyperinflammatory syndrome with multisystem involvement, on an immunopathological basis under the influence of a trigger factor which is a new coronavirus infection for MIS-C; for KD, a specific trigger has not been found yet.

In general, there is much in common between these two diseases, including many similarities in clinical phenotypes, and some patients with MIS-C fulfill the AHA criteria for KD (5). In both diseases, fever, mucocutaneous manifestations, conjunctivitis, hand and foot erythema/edema, and cervical lymphadenopathy are observed, but we noted that rash in MIS-C was more diffuse and extensive compared to KD (8, 9, 20, 22). In our research, these symptoms were also quite similar in both studied conditions. Patients with MIS-C were admitted to the hospital earlier than KD patients were, possibly due to raising concerns for the families the coronavirus presents. The main unifying sign of both diseases, except fever, mucocutaneous and lymph-node involvement, and systemic inflammation biomarkers, is the involvement of the heart and coronary arteries (9, 20, 22). Cardiac involvement in KD manifested with development of CA aneurisms, their subsequent thrombosis, and heart attack (5, 23). MIS-C is predominantly characterized by the acute myocardial injury with increased troponin I level and reversible dilatation of CA with rear CA thrombosis and heart attack (9, 11, 20, 22).

The main discriminative factors between two diseases are onset age, high CRP, thrombocytopenia, increased D-dimer, and GI involvement. MIS-C occurs in all age groups, but still the majority of patients are high school students, those with KD usually are younger, and 76% of them are children under 5 years old (24). The median onset age of the affected children of MIS-C in our study is 8.9 (5.3, 11.8) years, which is comparable to the previously published results where the median age ranges from 6 to 12 years (11, 22, 25, 26). However, it is important to emphasize that MIS-C can also occur in children under 1 year of age (27). Also, a hyperinflammatory condition similar to MIS-C in young adults after COVID-19 has been reported (28).

Diversity between the two illnesses is also obvious as there are signs and symptoms with high frequency in MIS-C which are less commonly or rarely presented in KD patients. For example, gastrointestinal symptoms are more common for MIS-C according to many studies including our observation (11, 22, 29). It should also be noted that among the studied patients with gastrointestinal disorders in our MIS-C group, one had acute appendicitis, which required laparoscopic appendectomy, and the “white” appendix was removed. Similar cases are also described in previously published works (30, 31). In addition, among significant differences between MIS-C and KD there are myocardial injury, hypotension/shock, and neurological disorders in MIS-C patients that are less frequent in KD (8, 20, 22, 32). Sixty-five percent of MIS-C patients admitted to ICU had CNS involvement and had serous meningitis and cerebral venous thrombosis as the most serious complications. Unless cardiac involvement can be presented in both diseases, there are some differences in the nature of cardiac findings. Thus, myocarditis is more common in MIS-C patients, while CA aneurisms are more typical for KD (9, 32–34).

Speaking of laboratory values, elevated inflammatory biomarkers were seen in both conditions in our study as well as in previously published articles (8, 20, 29). However, for example, CRP levels were significantly higher in MIS-C patients in our study, as well as in previously published articles (29). For assessment of cytokine storm syndrome, we applied HScore, which was created earlier for hemophagocytic lymphohistiocytosis. HScore >105 was associated with the severest signs of MIS-C: myocarditis [OR = 4.2 (95% CI: 1.5, 11.5), *p* = 0.005], pericarditis [OR = 7.1 (95% CI: 2.4, 21.6), *p* = 0.0003], shock [OR = 3.7 (95% CI: 1.4, 10.3), *p* = 0.009], and GI involvement [OR=5.4 (95% CI: 1.3, 22.3), *p* = 0.013]. HScore is a simple tool which might be used for assessment of severity of MIS-C. Additionally, there is a difference in platelet levels in MIS-C and KD patients in our study, with median of 185 and 520^*^10^9^/l, respectively. Thrombocytosis is typical for KD, but less common in MIS-C patients, who quite often have a tendency toward thrombocytopenia, especially at onset or during the peak of the disease (8, 20, 22, 29). Additionally, elevated biochemical markers of cardiac injury and highly elevated ferritin and D-dimer are typical for MIS-C patients (20, 22, 29). These findings seem to be less common for KD patients. For example, in our study median ferritin was 366 μg/l in the MIS-C group and 120 in the KD cohort, and D-dimer levels were also significantly different in studied conditions, 1,855 and 584 ng/ml, respectively. However, it is also reported that elevated D-dimer can actually be the risk factor of CA damage in KD patients (35).

MIS-C is close in manifestations to Kawasaki-shock syndrome as they are united by the fulminant development of heart injury, similar gastrointestinal symptoms, hyponatremia, and hypoalbuminemia (34). Factors causing the development of shock in KD are not fully understood, but an important role in its pathogenesis is played by more pronounced inflammation and especially intense and very rapidly developing vasculitis with thrombosis and endothelial damage (36). Probably, endothelial damage in MIS-C differs from KD, due to the unique characteristics of the pathogen SARS-CoV-2 and its affinity to the endothelium. The vascular wall damage due to COVID is primarily caused by the virus penetration into the cells of the vascular endothelium through the ACE2 receptor, and then after a certain period of time immuno-mediated endothelial damage develops (37).

The pathogenetic basis for both MIS-C and KD is the dysregulation of the innate immune response, and as a result in both diseases there is an excessive production of pro-inflammatory cytokines, up to a cytokine storm, which occurs much more frequently in MIS-C than in KD. Gruber et al. (38) detected the presence of autoantibodies in patients with MIS-C against not only endothelial but also gastrointestinal and immunocompetent cells, and obviously this is due to a number of pathophysiological and clinical differences. Neutralizing antibodies against SARS-CoV-2 can activate IL-18 and IL-16, myeloid chemotaxis, and activation of lymphocytes, monocytes, and natural killers, which lead to neutrophil activation, hypercoagulation, and thrombosis (38). These changes are not typical for children suffering from acute COVID-19, but characteristic for severe forms of COVID-19 in adults (39). NETosis on the basis of MIS-C in children and severe forms of COVID-19 in adults may provoke severe cytokine storm and inflammation leading to microthrombosis and irreversible involvement of the cardiorespiratory system (40). The main unresolved question is if MIS-C is a unique self-limited condition, related to COVID-19 infection, or it is the severest variant of KD triggered by the SARS-CoV-2 virus. The viral etiology of KD was previously described, and different types of coronaviruses (HCoV-NL63 and HCoV-NL229E) were mentioned as a possible cause of KD, but other studies did not confirm it (41–44).

### Study Limitations

The KMDscore has some limitations as well. First, it was developed using a retrospective study population, with the possibility of bias in the selection of this population. To minimize this bias and to ensure that no patient fulfilling our inclusion criteria was missed, we selected the study population by reviewing the medical records having non-bias diagnosis of MIS-C and KD. The historical nature of the KD cohort also restricted the efficacy of the results because it does not reflect the modern trends in KD in the time of the COVID-19 pandemic. The main difficulties are related to the absence of validated criteria of MIS-C.

## Conclusion

The DScore can be used to differentiate the diagnostic of having MIS-C from KD, accompanied with other diagnostic tests and procedures. Further investigations are required.

## Data Availability Statement

The raw data supporting the conclusions of this article will be made available by the authors, without undue reservation.

## Author Contributions

MK, LB, VC, VS, and YK contributed to the conception and design of the study. IA, ED, AM, EI, TK, OE, VM, LS, and OK organized the database. MK and IA performed the statistical analysis. MK, LB, and IA wrote the first draft of the manuscript, wrote sections of the manuscript, and had full access to all of the data in the study and take responsibility for the integrity of the data and the accuracy of the data analysis. MK, LB, IA, ED, AM, OE, EI, TK, VM, LS, VS, YK, OK, and VC have contributed equally to all of the following aspects: conception, acquisition of data, drafting, and revising the article. All authors contributed to the article revision, read, and approved the submitted version.

## Funding

This work was supported by the Russian Foundation for Basic Research (Grant No. 18-515-57001).

## Conflict of Interest

The authors declare that the research was conducted in the absence of any commercial or financial relationships that could be construed as a potential conflict of interest.

## Publisher's Note

All claims expressed in this article are solely those of the authors and do not necessarily represent those of their affiliated organizations, or those of the publisher, the editors and the reviewers. Any product that may be evaluated in this article, or claim that may be made by its manufacturer, is not guaranteed or endorsed by the publisher.
